# Development and Feasibility Assessment of a Sequential Antenna Deployment System Based on Fiber-Reinforced Shape Memory Polymer Composites

**DOI:** 10.3390/polym17202797

**Published:** 2025-10-20

**Authors:** Marylen T. De la Cruz, Riana Gabrielle P. Gamboa, Jon Dewitt E. Dalisay, Ricky Kristan M. Raguindin, Eduardo R. Magdaluyo

**Affiliations:** 1 Department of Mechanical Engineering, College of Engineering, University of the Philippines Diliman, Quezon City 1101, Philippines; rpgamboa1@up.edu.ph; 2 Department of Mining, Metallurgical and Materials Engineering, College of Engineering, University of the Philippines Diliman, Quezon City 1101, Philippines; rmraguindin1@up.edu.ph (R.K.M.R.);

**Keywords:** shape memory, antenna deployment, polymer composites, smart materials, thermal actuation

## Abstract

With the growing demand for reliable, low-impact deployment systems in small satellite missions, this work introduces an antenna deployment mechanism using fiber-reinforced shape memory polymer composites (SMPC). The mechanism utilized thermally activated SMPCs for stowage and release, configured with different glass transition temperatures (*T_g_*), tuned through the addition of poly(ethylene glycol) (PEG-600), for sequential actuation. The deployment mechanism consisted of three SMPC components with varying PEG concentrations: SMPC-P (0 wt%), SMPC-5 (5 wt%), and SMPC-10 (10 wt%). For component design, three bending angle configurations (BAC) of 20°, 30°, and 40° were tested. The samples exhibited the highest fixity ratio (93.58%, 95.76%, and 96.52% for SMPC-P, SMPC-5, and SMPC-10, respectively) when conformed to the 20° BAC. All samples achieved full recovery within 2 min, with PEG-incorporated composites exhibiting more uniform behavior across cycles, while recovery rates varied by material and BAC. Deployment testing confirmed the antenna was released successfully across all BACs. The 20° BAC exhibited the fastest response, completing deployment 24 s and 30 s ahead of the 30° and 40° BACs, respectively. The proposed mechanism exhibits promising potential for integration in future CubeSat missions. However, further testing under simulated space conditions is necessary to comprehensively assess and validate its performance.

## 1. Introduction

In recent years, the spread of space technology has been significantly influenced by the emergence of CubeSats. These miniaturized satellites have revolutionized the accessibility of space missions by being a cost-effective alternative to traditional satellites and by providing a platform for education [1]. Despite their compact size, CubeSats have shown notable applications in communications, Earth remote sensing, and biological research [2]. The technology has created opportunities for developing nations, particularly the Philippines, to establish their presence in space and address pressing national challenges. The successful implementations of the Diwata and Maya satellite programs serve as evidence of this progress. It has generated data necessary for coastal monitoring, agricultural planning, land use mapping, and disaster risk forecasting in the Philippines [3].

While CubeSats have driven significant progress, they are not without their challenges. One critical issue that has been consistently observed is the reliability of deployment systems, particularly concerning antennas [4]. Antennas are essential for receiving and transmitting signals during missions. They enable communication with ground stations and delivery of data for research and observation [5]. Given their critical importance, any failure in antenna deployment can severely compromise mission success [6]. Rivera and Stewart [7] examined 53 spacecraft failures associated with deployment issues. Their work revealed that antenna deployment failures accounted for 40% of all recorded anomalies, making it the second most prevalent deployment issue in spacecraft missions. One notable example is the 3U ExoCube satellite launched in 2015, which encountered an antenna deployment malfunction [8]. Failure analysis showed that the issue was associated with its burn wire release mechanism, specifically due to a break in the electrical pathway [9].

The burn wire mechanism is a release system that utilizes electrical current to sever a restraining wire that holds the deployable structure in place. This traditional method has been widely adopted for antenna deployment due to its simplicity, volume efficiency, and cost-effectiveness [10]. It has been implemented in various missions, including the Diwata 2 microsatellite’s ultra-high frequency (UHF) and very high frequency (VHF) antennas [11]. Despite its advantages, prior mission failures highlight the mechanism’s susceptibility to operational issues, including adverse effects of mechanical vibrations during launch, insufficient power supply, and wire harness interference.

Given these challenges, there is a need for a more reliable antenna deployment mechanism in CubeSat missions. Recent studies have explored innovative release mechanisms, including motorized systems [12], origami/kirigami folding techniques [13], gravity gradient methods [14], and shape memory material (SMM) actuation [15]. SMMs are an emerging class of intelligent materials that can be deformed into a temporary shape and returned to their original configuration when exposed to external stimuli such as heat, electricity, light, pH changes, humidity, or other environmental factors [16]. Compared to traditional deployment systems, SMMs exhibit slower actuation, which can be exploited for mitigating mechanical shock and vibrations transmitted during antenna deployment.

Shape memory polymer composites (SMPCs) are a class of SMMs that integrate the programmable actuation properties of shape memory polymers (SMPs) with the enhanced mechanical strength provided by fiber reinforcements [17]. Among all smart materials, this hybrid composition offers a combined structural and functional advantage due to its lightweight nature, high recovery force, improved stiffness, and cost-effectiveness [18]. Recent years have seen a rise in research investigating the suitability and performance of SMPCs in aerospace applications. For instance, Moskvichev and Larichkin [19] developed a carbon fiber-reinforced (CF) polyurethane-based SMPC for space antenna reflector frames and analyzed its functional and mechanical properties. Results showed that the SMPC achieved an optimal shape recovery rate and degree, highlighting its potential for applications requiring thermal insulation. Similarly, Jang et al. [20] investigated how prolonged subjection to simulated low Earth orbit (LEO) conditions affects the mechanical behavior of CF SMPCs over time. Using the time–temperature superposition principle, the study predicted that CF SMPCs would experience only minor changes in shape memory performance after accelerated exposure to harsh conditions, including high vacuum, atomic oxygen, and ultraviolet (UV) light. These findings suggest that SMPCs can maintain their function and performance under LEO conditions and are suitable for CubeSat missions, which are typically deployed in this environment [21].

While prior studies have demonstrated the utility of SMPCs for hinges [22], solar arrays [23], and reflector antennas [24], their application in programmable multi-stage deployment systems remains largely unexplored. The knowledge gap lies in creating a coordinated actuation sequence from multiple SMPC components without complex control systems. This study addresses this gap by developing and validating a novel sequential deployment mechanism for antennas. Epoxy-based CF SMPC components were fabricated and arranged to stow an antenna and release it slowly given a specified thermal environment. The deployment sequence is programmed directly into the system by tuning the glass transition temperature of each SMPC component through chemical modification. This approach enables a controlled, low-impact, and power-free antenna release, showcasing an alternative deployment mechanism for CubeSats.

The goal is to address problems with traditional deployment mechanisms: burn wire mechanisms are prone to electrical failures and generate significant mechanical shock upon release, spring-loaded mechanisms also induce strong shocks that can damage sensitive payloads, and motorized mechanisms result in increased mass, complexity, and power. The proposed SMPC-based mechanism mitigates these issues by enabling a controlled low-shock deployment, passively actuated by the space thermal environment. Table 1 provides a qualitative comparison of these technologies.

## 2. Materials and Methods

### 2.1. Proposed Deployment Mechanism

**Figure 1:**
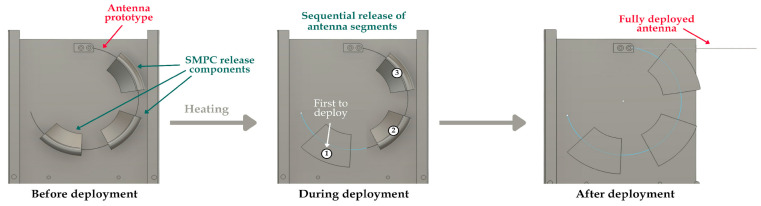
Schematic illustration of the proposed antenna deployment, shown here with a bending angle of 30°. The numbers indicate the order of deployment.

A proposed deployment mechanism, as shown in Figure 1, was conceptualized to feature three SMPC components, each securing alternating segments of the antenna. In its stowed state, the components maintained a pre-programmed “curled” configuration to restrain the antenna. Upon heating above their respective glass transition temperatures (*T_g_*), the components underwent an uncurling transition toward a flat, rectangular shape, thereby initiating release. To enable low-impact deployment, the three components were tailored to achieve incrementally higher *T_g_* values through modified composition and curing procedures, thus making their activations sequential. This design resulted in a controlled, slow deployment of the antenna in response to environmental heating. The mechanism was further evaluated across three bending-angle configurations to determine the best design for reliable deployment.

**Figure 2:**
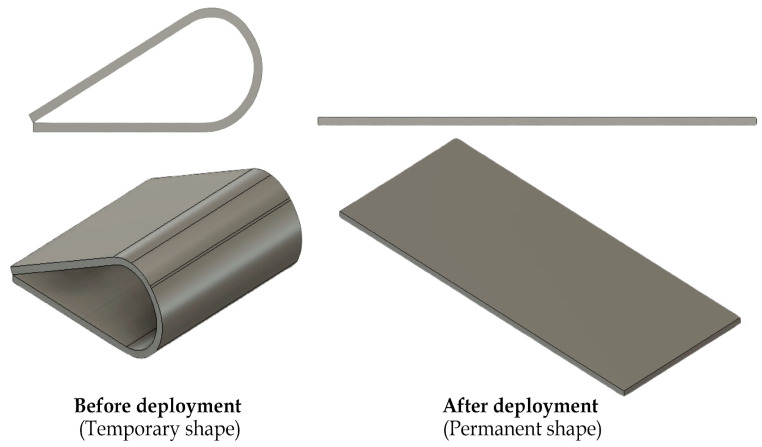
Pre- and post-deployment configurations of the SMPC release components.

The temporary and permanent configurations of each component are illustrated in Figure 2, where the curled geometry represents the restrained state prior to deployment, and the flattened geometry shows the recovered state after activation above the glass transition temperature.

### 2.2. Shape Memory Polymer (SMP) Fabrication

A baseline epoxy matrix was first prepared together with two PEG-modified test formulations. The matrix consisted of a commercial epoxy system, Polytech Laminating Epoxy (Polymer Products (Phils.) Inc., Pasig City, Philippines), composed of Bisphenol A diglycidyl ether (DGEBA) resin and a modified amidoamine hardener in a 2:1 volumetric ratio. The reinforcing material was provided by a hybrid 2 × 2 twill woven fabric Carbon Fiber Twill, TN-07-T, (Polymer Products (Phils.) Inc., Pasig City, Philippines) composed of aramid (primary fiber) and carbon 3k (secondary fiber) in a 0°/90° fiber orientation. A fixed fiber-to-resin mass ratio of 15:85 was maintained for all samples [25]. Polyethylene glycol (PEG-600) [26] was employed as a plasticizer at concentrations of 5% and 10 wt%. Relative to the total matrix volume, yielding three formulations: (1) neat SMP (SMPC-P), (2) SMP with 5 wt% PEG (SMP-5), and (3) SMP 10 wt% PEG (SMP-10).

For sample preparation, the resin and hardener were first preheated to 40 °C in a water bath to reduce viscosity for a more effective mixing in the prescribed ratio. The components were manually stirred with a glass rod for 1 min, then on a digital vortex shaker (Thermo Fisher Scientific Inc., Waltham, MA, USA) for 1 min. For PEG-modified systems, the PEG was initially mixed with the resin in the water bath prior to the incorporation of the hardener. To minimize the presence of bubbles, all mixtures were degassed in an ultrasonicator at room temperature for 13 min.

### 2.3. SMPC Fabrication and Curing

The epoxy-based matrices were incorporated into the fibers via the hand lay-up method. The hand lay-up method was chosen for its suitability in fabricating proof-of-concept prototypes. For fabricating flight hardware in future work, this process would be improved using more controlled and repeatable manufacturing techniques such as vacuum-assisted resin transfer molding. For each formulation, a rectangular mold (15 mm × 30 mm) and flat metal sheets were prepared and coated with mold release wax to prevent adhesion during curing. Half of the total matrix volume was first dispensed onto the mold surface and evenly distributed with a 3D-printed scraper. A 15 mm × 30 mm fiber sheet was then placed over the resin layer and spread uniformly in all directions to promote thorough impregnation and minimize entrapped air. The remaining half of the matrix was subsequently poured over the fiber sheet, spread evenly, and covered with a flat metal sheet to ensure uniform distribution and consistent specimen thickness. The composites were then subjected to thermal curing in a DKN402 constant-temperature oven (Yamato Scientific Co., Ltd., Tokyo, Japan) using a three-step cycle: (i) 2 h at 80 °C, (ii) 2 h at 100 °C, and (iii) 1 h at 120 °C [27]. After curing, the specimens were cut to final dimensions of 30 mm × 78 mm using industrial-grade shears.

### 2.4. SMPC Programming

**Figure 3:**
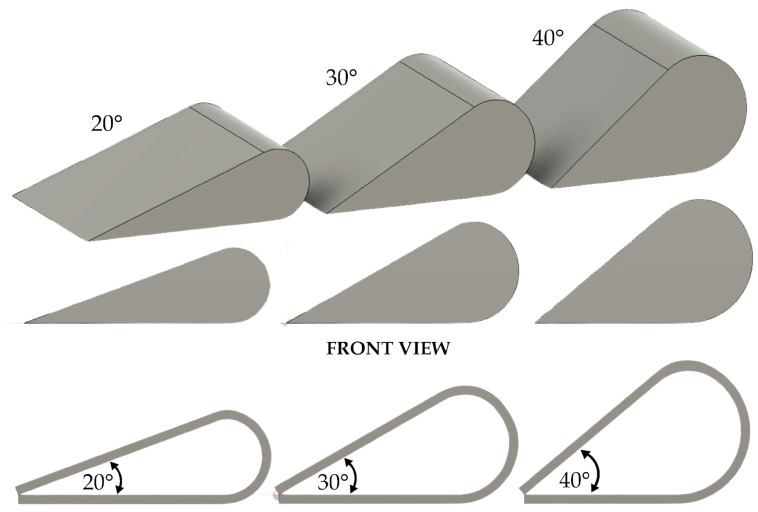
Configurations of the 3D printed molds for SMPC programming and bending angle configurations.

To obtain the desired geometries for the proposed deployment mechanism, molds were 3D printed using acrylonitrile butadiene styrene (ABS) with bending angle configurations of 20°, 30° and 40°, as shown in Figure 3.

For programming, each SMPC variant was heated to approximately 30 °C above its respective glass transition temperature and conformed to the target configuration using the 3D-printed molds. To maintain uniformity, the samples were secured in a vice during forming. After cooling to room temperature, the programmed samples were removed from the molds and prepared for subsequent testing.

For this feasibility study, a single specimen was characterized for each condition. While sufficient for demonstrating the proof-of-concept, a larger sample size would be required for a full statistical validation of the results.

### 2.5. Shape Memory Behavior

**Figure 4:**
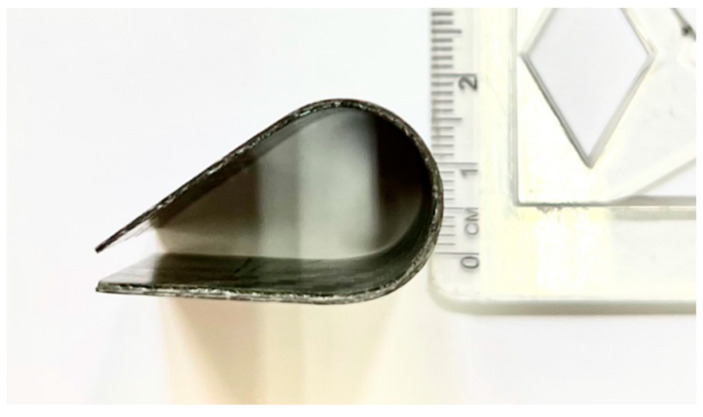
SMPC sample subjected to a 40° BAC.

The shape memory performance of the fabricated samples was evaluated at the macroscale, following the methodologies outlined by Margoy et al. [28] and Bellisario et al. [29]. To ensure consistency, each SMPC sample underwent thermal activation for 2 min followed by 2 min of cooling at room temperature; this is considered as one thermomechanical cycle. Each sample completed five cycles under bending angles of 20°, 30°, and 40°. Figure 4 shows a visual presentation of the SMPC subjected to a 40° BAC.

#### 2.5.1. Shape Fixity Ratio, R_f_

Shape fixity ratios were evaluated based on the radius of curvature, *r*, and the corresponding bending curvature, *k*, values of the specimens, where bending curvature was calculated using the relation:

(1) $$k=\frac{1}{r}$$

This parameter was then used in the shape fixity ratio (*R_f_*) formula, as defined in the study by Kumar et al. [30], and calculated using the following equation:

(2) $$R_{f}\left(\%\right)=\frac{k_{nl}}{k_{p}}\times 100\%$$

where *k_nl_* represents the bending curvature measured under no-load conditions, prior to thermal activation, and *k_p_* refers to the programmed or ideal bending curvature defined by the mold.

To quantify the shape fixity ratio, the formed shape of the SMPC sample for each thermomechanical cycle was outlined on paper, and the height and length were measured in millimeters to establish a scale. The ideal or programmed shape from the mold was also outlined for comparison. These outlined configurations were then scanned and analyzed using ImageJ 1.54p. After calibrating the scale using the recorded dimensions, a circle was fitted to the bending region to determine the curvature, and the corresponding radius of curvature was extracted for further analysis.

#### 2.5.2. Shape Recovery Ratio, R_r_

For this application, only the relative shape-recovery ratio (relative *R_r_*) was obtained due to the difficulty of measuring the sample’s bending curvature. The relative recovery ratio was quantified using the following equation:

(3) $$R_{r}\left(t\right)=\frac{\theta \left(t\right)\;-\;\theta_{start}}{\theta_{full}\;-\;\theta_{start}}\;\times \;100\%$$

where *θ_start_* corresponds to the angle measured immediately before recovery, *θ_full_* corresponds to the angle measured after full recovery, and *θ(t)* corresponds to the angle measured at time *t*. A shape recovery ratio of 100% indicates complete recovery to the original programmed shape.

**Figure 5:**
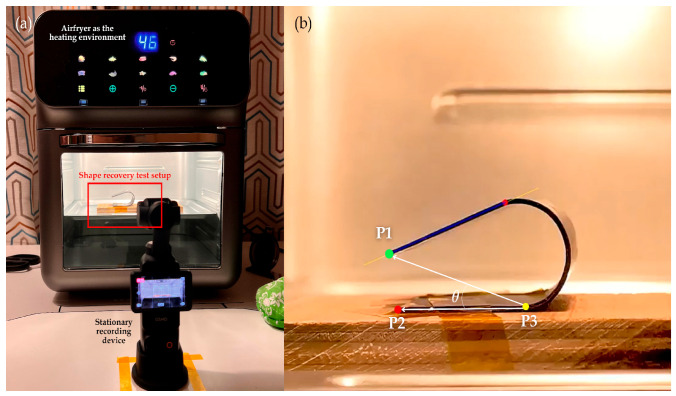
Experimental setup for shape recovery test (**a**) and a sample frame from annotated video used for recovery tracking (**b**).

As shown in Figure 5a, the experimental setup used to evaluate the recovery behavior of each sample involved recording every thermomechanical cycle with an Osmo Pocket 3 camera (SZ DJI Technology Co., Ltd., Shenzen, China) at 4K resolution and 30 frames per second. The footage was post-processed in Premiere Pro 2025 (Adobe Inc., San Jose, CA, USA) to stabilize the video and maintain consistent scaling.

Each video was then analyzed using a custom Python 3.13 script, in which three key points were manually plotted: (i) at the tip of the unraveling arm (P1), (ii) at the tip of the fixed arm (P2), and (iii) another point on the fixed arm of the sample (P3). Figure 5b illustrates a sample frame from the annotated video. For each frame, three reference points were tracked to determine the instantaneous angle subtended by the points throughout the recovery process. The resulting *x* and *y* coordinates of the tracked points were exported and further processed in Excel 2025 (Microsoft Corporation, Redmond, WA, USA). Using the geometric relationship between the three points, the subtended angle was obtained. Lastly, the shape recovery ratio was plotted over time to visualize each sample’s recovery progression.

### 2.6. Function Validation Through Deployment Testing

**Figure 6:**
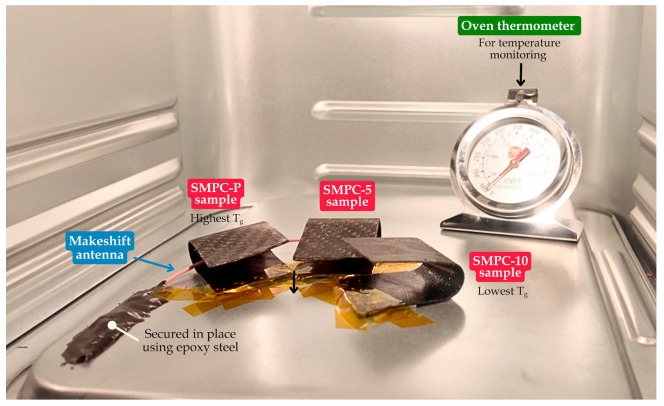
Experimental setup for antenna deployment testing.

Antenna deployment tests for the SMPC proof-of-concept were conducted using nine samples, divided into three trials, each corresponding to bending angles of 20°, 30°, and 40°. The deployment sequence is designed to initiate near the *T_g_* of the most sensitive component (SMPC-10, ~60 °C) and complete after the *T_g_* of the component without PEG addition is exceeded (SMPC-P, ~90 °C). For each trial, samples were placed inside an oven starting at 60 °C, with setpoint increments of 10 °C every 30 s until full deployment was achieved. This is significantly faster than the expected thermal ramp rate in orbit (typically <5 °C per minute), and further testing under simulated space conditions is required for full validation. A metal wire rod, chosen for its ability to spring back to its original shape after deformation, served as a surrogate for a CubeSat antenna, simulating the stored strain energy and recovery force of a stowed antenna. The setup consisted of a metal pan housed within an air fryer oven (OOKAS, Metro Manila, Philippines), with the antenna prototype bonded to it using epoxy steel. Figure 6 shows the experimental setup for antenna deployment testing.

A 10 cm by 10 cm section was designated as the deployment workspace inside the thermal chamber. In each trial, one sample of SMPC-P, SMPC-5, and SMPC-10 was configured to the same bending angle and attached to the pan using double-sided and heat-resistant tapes, with SMPC-P positioned closest to the wire and SMPC-10 the farthest, thereby enabling sequential activation. All tests were documented using an Osmo Pocket 3 camera (SZ DJI Technology Co., Ltd., Shenzen, China) at 4K resolution and 30 fps, providing the data for analysis. Figure 6 illustrates the experimental setup for the antenna deployment testing.

Testing included both qualitative and quantitative assessments, as detailed in Table 2 and Table 3, respectively. Table 2 highlights four key qualitative observation criteria: (i) deployment success, (ii) motion quality, (iii) deployment completeness, and (iv) structural inspection. The quantitative measurements, on the other hand, include deployment such as deployment time and activation temperature, as indicated in Table 3.

## 3. Results and Discussions

### 3.1. Shape Fixity Ratio

**Figure 7:**
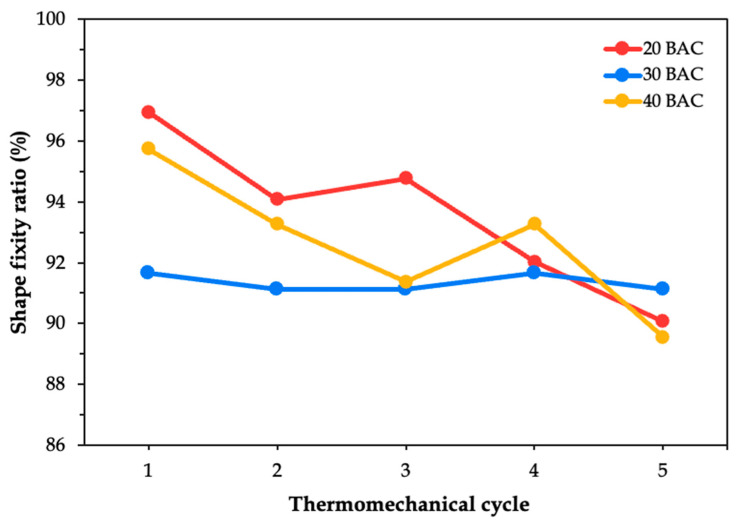
Shape fixity ratio of SMPC-P at three BAC levels across five thermomechanical cycles.

The shape fixity ratio was evaluated based on the bending curvature estimation. It determines the SMPC’s ability to retain its deformed shape after unloading. The fixity performance of each SMPC case was plotted across all five thermomechanical cycles. Figure 7 illustrates the behavior of the unmodified composite SMPC-P after being subjected to three different bending angle configurations. It can be observed that as the cycles progressed, the shape fixity of SMPC-P generally decreased. This trend was consistent across the 20° and 40° bending angle configurations. However, for the 30° configuration, the fixity ratio appeared relatively flat rather than showing a steady decline, which may suggest more stable fixity behavior at this intermediate angle.

During the first thermomechanical cycle, the SMPC-P specimen exhibited its maximum shape fixity at 20° BAC (96.95%), followed by 40° (95.74%), and then 30° (91.67%). However, by the fifth cycle, the highest fixity was observed at 30° (89.55%) while the lowest was at 40° (89.55%). This shift suggests that with continued cycling, SMPC-P may gradually lose its ability to maintain tighter bending configurations. Previous studies have shown that SMPCs typically maintain shape fixity ratios above 95%, even after more than 30 thermomechanical cycles, with minimal signs of degradation [31]. In contrast, the present study observed a decline in the shape fixity within only five cycles, with the 20 BAC and 40 BAC systems showing reductions of 7.10% and 6.47%, respectively. This discrepancy may be attributed to the differences in the epoxy system used or to the loading configurations to which the SMPC was subjected.

**Figure 8:**
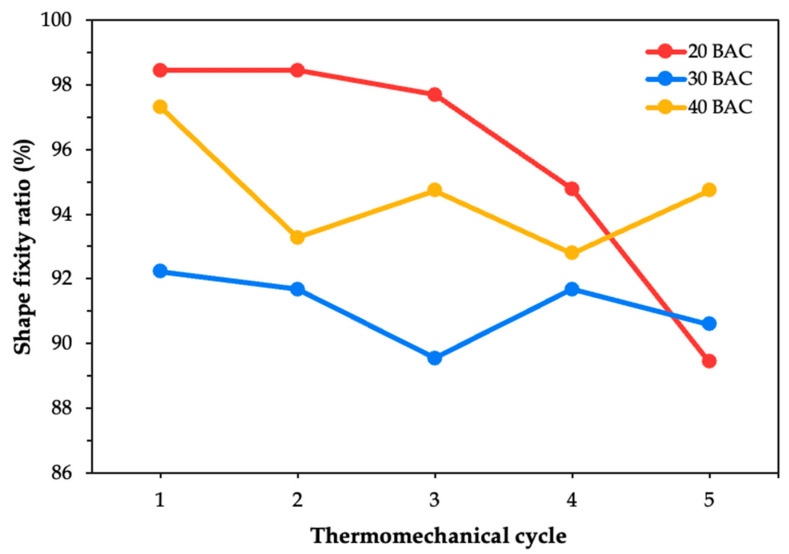
Shape fixity ratio of SMPC-5 at three BAC levels across five thermomechanical cycles.

The shape fixity ratio for the first cycle of SMPC-5 across all three BACs increased compared to that of SMPC-P, as evidenced by Figure 8. This improvement results from the addition of PEG, which increases the chain mobility of the SMPC during the programming phase. In effect, the reduced viscosity allows the material to conform more effectively to the mold geometry and retain the deformed shape upon cooling [32]. Similar findings were reported by Jayan et al. [33] where PEG-modified composite systems showed improved flexibility and reduced residual stress in the epoxy matrix. The 20° BAC exhibits a gradual but non-linear decline, with sharper drops observed during the later cycles. In contrast, for the 30° and 40° BACs, the fixity ratio fluctuated over time, where they were initially increasing before eventually decreasing. This difference can be attributed to the higher mechanical strain required to achieve tighter curvatures at lower bending angles like 20° BAC. Moreover, this also suggests that even though the softer matrix brought by the PEG may delay the onset of degradation, it cannot entirely prevent it over repeated cycles.

**Figure 9:**
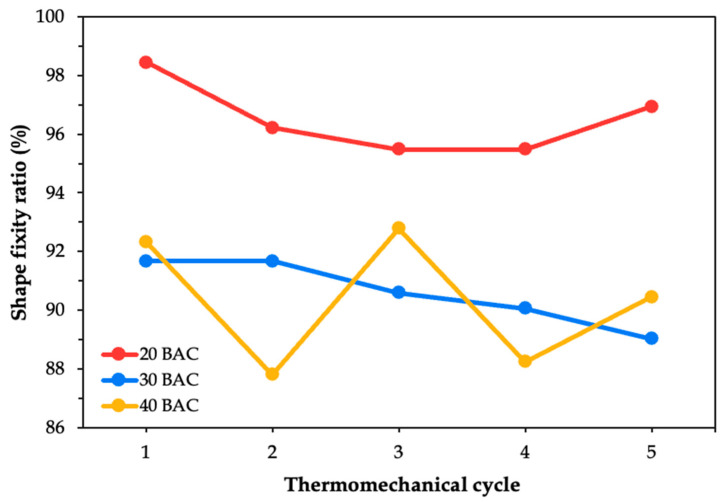
Shape fixity ratio of SMPC-10 at three BAC levels across five thermomechanical cycles.

The SMPC-10 samples exhibited irregular trends across the five thermomechanical cycles, as shown in Figure 9. It can be observed that the 20° BAC, which imposed the highest programming strain, consistently yielded the highest shape fixity ratio. The resulting curve depicts a shallow convex shape, which suggests reorganization of the polymer chains and improved stress accommodation after the initial cycles. Moreover, this behavior can also be linked to the presence of 10 wt% PEG-600, which softened the current epoxy matrix and enhanced its flexibility.

For the 30° BAC, the shape fixity ratio exhibited a steady, gradual decline over the five cycles, whereas the 40° BAC displayed a fluctuating trend, consistent with the behavior previously observed in SMPC-5. The fixity values for 40° BAC rise and fall inconsistently over the five cycles, indicating unstable shape retention. These findings suggest that the bending angle and programming strain strongly influence long-term fixity performance of the fabricated SMPCs, with smaller bending angles and higher strains (20° BAC) providing more reliable stability. This is consistent with the results of Li and Xu [34] who reported that higher prestrain levels and longer relaxation times led to significantly improved shape fixity ratios, with fixity reaching up to 73% at 30% prestrain.

**Figure 10:**
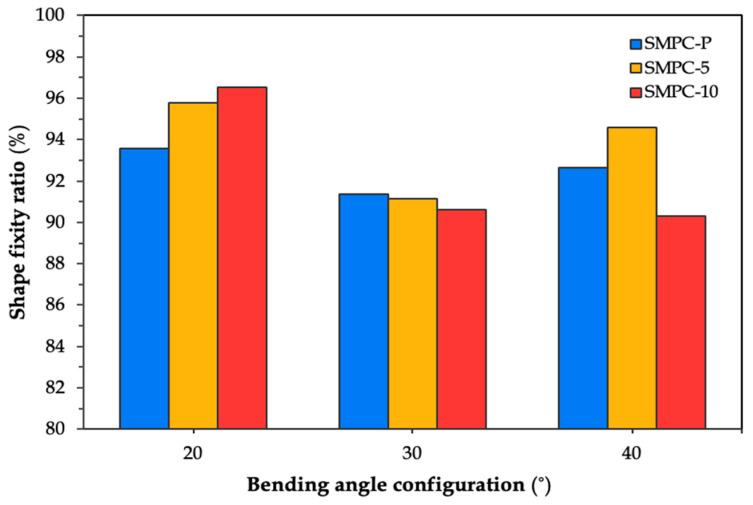
Average shape fixity ratio of SMPC formulations across three BAC levels.

Figure 10 compares the average shape fixity ratios of the three SMPC formulations across all bending angle configurations.

It can be observed that all three SMPC formulations obtained their highest fixity values when conformed to the 20° BAC, with values of 93.58%, 95.76%, and 96.52% for SMPC-P, SMPC-5, and SMPC-10, respectively. This is supported by the fact that tighter bending angles require greater stress to deform the sample, which in turn helps the material more firmly retain its temporary shape after cooling. The next highest fixity values were observed at the 40° BAC, while the lowest were recorded at the 30° BAC. Among the three formulations, SMPC-10 exhibited the highest overall fixity performance, which, as previously discussed, can be attributed to the improved matrix flexibility and chain mobility during deformation provided by the added PEG.

The fixity of shape memory polymer materials depends on their ability to immobilize polymer chains once cooled below the *T_g_*. The incorporation of PEG-600, with its flexible ether linkages and plasticizing effect, into the epoxy network increases chain mobility and reduces *T_g_*, thereby making the matrix softer and more flexible during the programming phase. The enhanced molecular mobility provided by PEG-600 promotes faster stress relaxation and improved viscoelastic recovery upon load removal. Specifically, PEG-600 acts as a plasticizer by intercalating between epoxy chains. Its flexible, linear molecules disrupt the close packing of the polymer network, increasing the spacing between chains. This dilution of reactive sites lowers the crosslink density, thereby increasing free volume and chain mobility. As a result, less energy is required for molecular motion, which manifests as a reduced glass transition temperature [35].

A separate research article by the same authors focused on the material development and characterization of the SMPC discussed in this paper, where dynamic mechanical analysis (DMA) confirmed the decreasing *T_g_* trend. This reduction in *T_g_* was further supported by the improved fixity ratios and smoother recovery curves observed in SMPC-5 and SMPC-10, as presented in the succeeding discussions.

### 3.2. Shape Recovery Ratio

The shape recovery ratio was evaluated to determine the SMPC’s ability to return to its original shape after thermal activation. The recovery behavior of each sample was recorded and plotted as a function of time, with all five thermomechanical cycles overlaid to observe the evolution of recovery performance across cycles.

**Figure 11:**
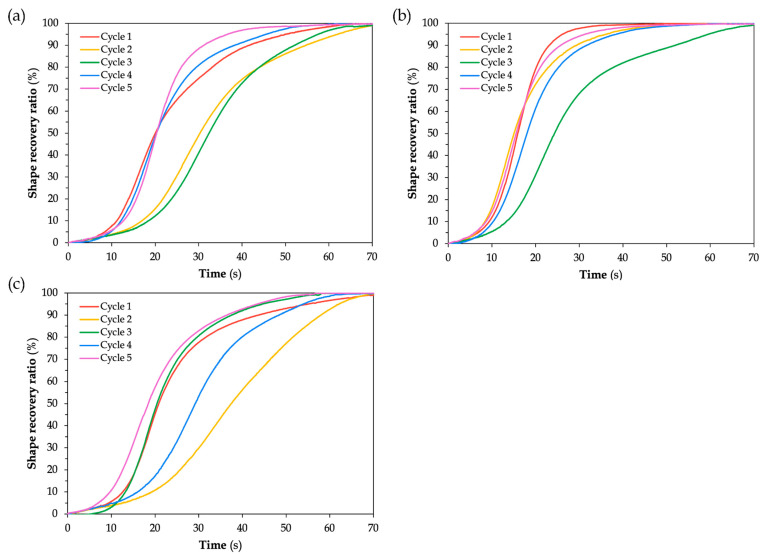
Shape recovery ratio of SMPC-P subjected to (**a**) 20° BAC, (**b**) 30° BAC, and (**c**) 40° BAC.

Figure 11 shows the shape recovery ratio plots of SMPC-P under the 20°, 30°, and 40° bending angle configurations. Across all plots, the recovery profiles exhibit a sigmoidal form, which is characteristic of shape memory behavior. According to Lei et al. [36], the shape memory behavior is characterized by three phrases: (i) an initial lag phase with minimal recovery, (ii) a rapid recovery phase marked by a sharp increase in recovery ratio, and (iii) a final plateau where the SMPC stabilizes near its original configuration.

For the unmodified SMPCs, it can be observed that as the bending angle increased, the separation between the recovery curves became more noticeable, particularly at the 40° BAC. This behavior indicates greater variability in recovery performance across cycles. Despite this, all samples were able to achieve a 100% shape recovery ratio in all five cycles. The fastest recovery cycle varied depending on the BAC. For the 20° BAC, cycles 4 and 5 exhibited similar recovery times. For the 30° BAC, cycle 2 was the earliest to initiate recovery, but cycle 5 reached 100% the fastest. Lastly, for the 40° BAC, cycle 1 was the first to reach full recovery, followed closely by cycles 3 and 5.

This suggests that while SMPC-P achieves full recovery across all bending angles, its recovery response becomes less consistent with increasing bending angle configuration. Ideally, shape recovery performance becomes more stable as the number cycles progress because of molecular alignment and stress relaxation. However, for unmodified SMPCs, such as SMPC-P, the absence of a softening agent like PEG can lead to microstructural fatigue over time, resulting in variability in recovery speed, even if full shape recovery is eventually achieved.

A similar study was conducted by Li et al. [25] who examined the recovery performance of unidirectional carbon fiber-reinforced SMPCs with varying mass fiber fractions. All composite formulations achieved recovery ratios exceeding 93% at 120 °C, with complete recovery obtained after 20 min. Although their SMPCs exhibited higher glass transition temperatures and were tested under different thermal conditions, the overall recovery trends were consistent with those observed in this work. Furthermore, their observation that loss factors and stiffness losses decreased significantly during the first three cycles, but eventually stabilized after ten cycles, helps explain the early-cycle variability and the gradual stabilization of recovery performance across the thermomechanical cycles in this study.

**Figure 12:**
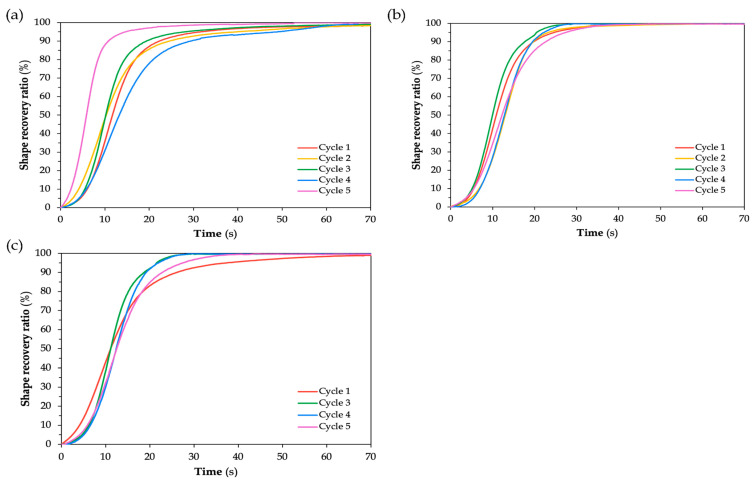
Shape recovery ratio of SMPC-5 subjected to (**a**) 20° BAC, (**b**) 30° BAC, and (**c**) 40° BAC.

**Figure 13:**
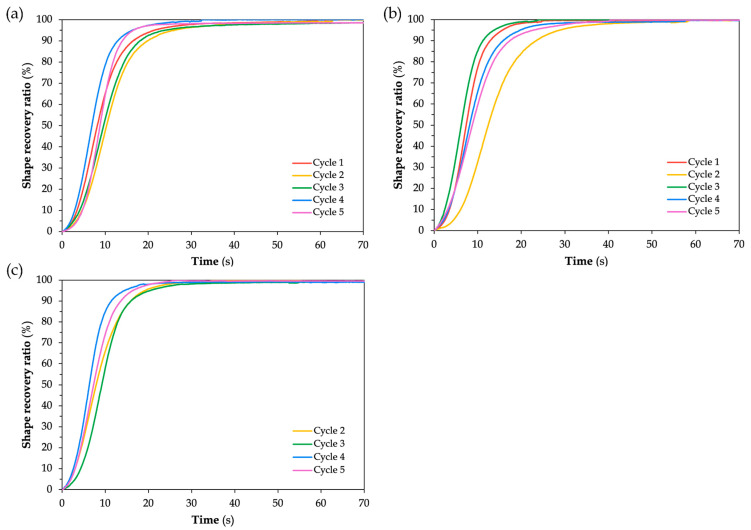
Shape recovery ratio of SMPC-10 subjected to (**a**) 20° BAC, (**b**) 30° BAC, and (**c**) 40° BAC.

For Figure 12 and Figure 13, which correspond to SMPC-5 and SMPC-10, respectively, the recovery curves across all five cycles appear more tightly clustered compared to the broader spread observed in SMPC-P. This suggests that the addition of PEG may have contributed to improved and uniform recovery performance across all cycles and BACs. It is important to note that Cycle 1 for SMPC-10 at 40° BAC is absent from the plot due to video instability during testing where continuous motion and image blurring prevented the collection of reliable data. Likewise, Cycle 2 for SMPC-5 at 40° BAC was excluded from the analysis as its recovery behavior deviated significantly from the other cycles due to an experimental inconsistency during that trial.

For all BACs in SMPC-5 (see Figure 12), the samples were able to reach 100% shape recovery, particularly those subjected to 30° and 40° BACs. However, the sample at 20° BAC took a longer time to stabilize. This may be due to the higher strain energy stored during programming at lower angles, which influences the rate of recovery despite achieving full recovery eventually. At 20° BAC of SMPC-5, Cycle 5 exhibited a noticeably faster recovery compared to the other cycles. This aligns with the expected behavior of SMPCs, where repeated thermomechanical cycling gradually improves recovery speed as the material adapts and becomes more responsive through internal structural adjustments. This is also evident in the SMPC-10 samples, where Cycle 4 exhibited the fastest recovery at 20° BAC, and Cycles 4 and 5 showed the quickest responses at 40° BAC.

**Figure 14:**
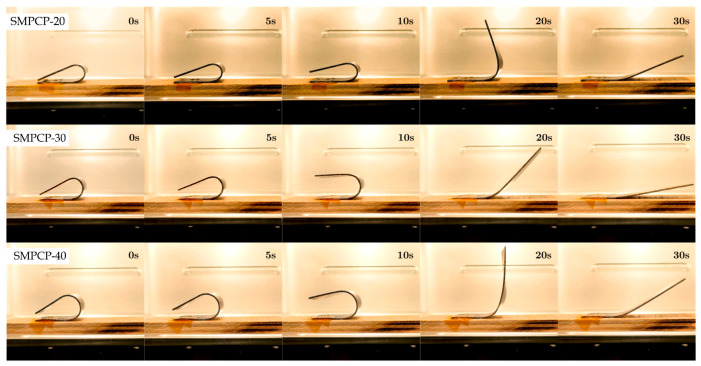
Shape recovery progression of SMPC-P under three different BAC conditions, recorded during the 5th thermomechanical cycle.

**Figure 15:**
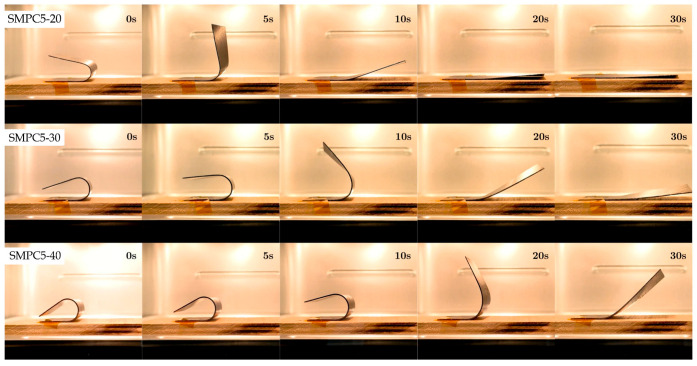
Shape recovery progression of SMPC-5 under three different BAC conditions, recorded during the 5th thermomechanical cycle.

**Figure 16:**
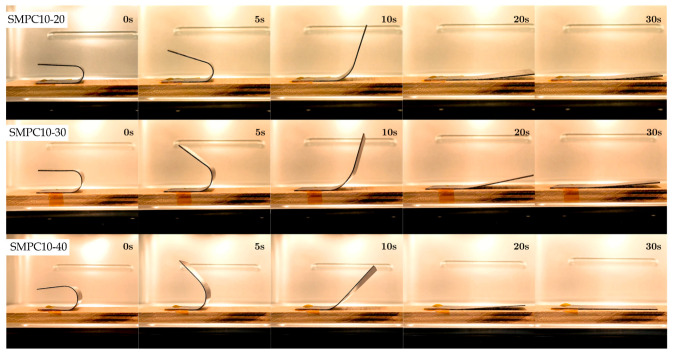
Shape recovery progression of SMPC-10 under three different BAC conditions, recorded during the 5th thermomechanical cycle.

Figure 14, Figure 15 and Figure 16 show snapshots of the shape recovery behavior. The recovery responses for each SMPC formulation during the fifth thermomechanical cycle are presented to show recovery performance given accumulated cyclic effects. For SMPC-P, the fastest recovery response was observed in the 30° BAC configuration, followed by 40° and then 20°. In contrast, SMPC-5 exhibited the quickest recovery under 20° BAC, followed by 30° and 40°. Finally, for SMPC-10, the sample subjected to 40° BAC recovered the fastest, followed by 20° and then 30°. This variation in recovery performance across formulations and bending angles highlights the importance of conducting further research to better understand the interactions between SMPC composition, programming strain, and recovery kinetics.

### 3.3. Function Validation Through Deployment Testing

**Figure 17:**
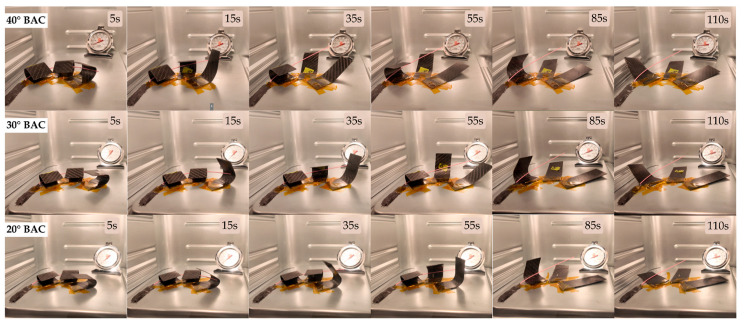
Deployment progression of the antenna for 20°, 30°, and 40° bending angle configurations.

The functional performance of the SMPC-based antenna deployment mechanism was first evaluated through a qualitative assessment based on four key criteria: deployment success, motion quality, deployment completeness, and structural cracks. Figure 17 illustrates the deployment mechanism over time for the different bending angle configurations.

Table 4 summarizes the observations gathered, providing insights into the consistency of the deployment mechanism across the various trials and the potential limitations associated with each variable.

Across all bending angle configurations, all SMPC samples successfully deployed and were able to release the antenna from its stowed position, demonstrating consistency in the basic activation and recovery behavior of the shape memory polymer composites. However, motion irregularities were observed, particularly with the SMPC-10 sample, which consistently exhibited partial jitteriness during the initial release phase. In contrast, the second and third samples (SMPC-5 and SMPC-P) deployed more smoothly. This behavior may be attributed to uneven heating inside the oven, leading to non-uniform temperature distribution, especially critical for SMPC-10, which is more heat-sensitive due to its higher PEG content and lower glass transition temperature. Moreover, its early softening may have reduced its ability to firmly hold the spring-back antenna, resulting in less controlled motion. This aligns with the trends reported by Singh et al. [37], who investigated PEG as a plasticizer and showed that epoxy–PEG formulations exhibited improved tensile strength, modulus, and failure strain at moderate PEG-400 concentrations (≤10 wt%), while higher PEG contents induced over-plasticization, which led to excessive matrix softening. They also noted that increased PEG content reduced flexural stiffness and strength under three-point bending due to greater polyol domain formation. Furthermore, prior reports indicate that higher molecular weight PEGs, such as PEG-600 used in this study, may further reduce crosslinking density and increase flexibility, which could amplify this effect. Although the present deployment setup is not a standard flexural test, the uneven edge deployment observed is consistent with this mechanism, where excessive PEG loading lowers rigidity and hinders recovery uniformity. Future iterations could mitigate this jittery motion by adjusting the PEG concentrations to balance stiffness and *T_g_*, or modifying the SMPC-10 component’s geometry to provide stiffness without changing its *T_g_*.

Nevertheless, all antennas ultimately achieved full deployment, indicating functional reliability despite the initial jitteriness. No visible cracks were found on any of the specimens after deployment, suggesting that they can withstand the mechanical load and force imposed by the antenna. However, it must be noted that these findings reflect short-term performance only. To validate reliability and repeatability over time, further testing under longer time frames and multiple deployment cycles is recommended.

Table 5 shows the relationship between bending angle, deployment time, and thermal activation. At a bending angle of 20°, deployment occurred in approximately 1 min. 30 s, despite the increase in bending angle. However, deployment times at 30° and 40° were longer, at 116 s and 122 s, respectively. This suggests that an increase in bending angle results in a measurable delay in antenna deployment. At 40°, the deployment time increased to 1.3 times that of the 20° configuration, likely attributed to the larger bending stress experienced by tighter bends leading to more strain energy [37] and possibly resulting in stronger recovery forces. Despite this, all samples activated promptly once the temperature reached 60 °C, indicating consistent sample fabrication and effective modification of the material’s glass transition temperature. Overall, bending angle configuration at 20° provided the best balance between deployment rate while maintaining structural integrity. It had the fastest shape recovery rate among all angles. Notably, it also had the best fixity ratio, meaning it would retain its deformed shape more effectively prior to activation, an essential factor for ensuring reliable deployment after prolonged storage or transportation.

Overall, bending angle configuration at 20° provided the best balance between deployment rate while maintaining structural integrity. It had the fastest shape recovery rate among all angles. Notably, it also had the best fixity ratio, meaning it would retain its deformed shape more effectively prior to activation, an essential factor for ensuring reliable deployment after prolonged storage or transportation.

## 4. Conclusions

This work proposed and validated a low-impact CubeSat antenna deployment mechanism using epoxy-based SMPCs reinforced with carbon-aramid fibers. Shape memory behavior testing revealed that shape fixity was highest at 20° BAC across all SMPC types, which could be attributed to the higher stress imposed by the tighter curvature, enabling improved shape retention after cooling. Notably, SMPC-10 showed the best overall fixity at 96.52%. All samples achieved near to full recovery, with PEG-modified SMPCs showing more uniform and consistent performance across cycles. Deployment experiments validated the functionality of the proposed mechanism, with all SMPC samples successfully deploying antennas at 20°, 30°, and 40° BACs. The 20° configuration demonstrated the fastest deployment time (92 s), while the 40° configuration was the slowest (122 s). SMPC-10 samples exhibited minor motion irregularities across all BACs, likely due to earlier softening resulting from its lower *T_g_*. All samples activated reliably at 60 °C, and no structural cracks were observed post-deployment, indicating mechanical integrity. These results establish SMPCs as a lightweight, power-free, and reliable alternative for CubeSat antenna deployment. Future work should explore alternative bending angles to improve deployment time and stress distribution and further validation is required under simulated space conditions. Furthermore, future work must involve testing with flight hardware, including actual CubeSat antennas, to fully validate the system’s performance and interaction with a realistic deployable structure. The next phase of our research will focus on thermal vacuum chamber (TVAC) testing to assess deployment reliability under vacuum given thermal cycles, simulating LEO conditions.

## Figures and Tables

**Table 1:** Comparison of traditional deployment mechanisms with the proposed SMPC-based mechanism.

Feature	Burn Wire	Spring- Loaded	Motorized	Proposed SMPC-Based
Deployment Shock (Lower is better)	High	High	Low	Low
Power Consumption (Lower is better)	Low	None	High	None
Mass & Complexity (Lower is better)	Low	Low	High	Low
Deployment Speed (No preference)	Fast	Fast	Slow	Slow
Reliability (Higher is better)	Moderate	High	High	Needs testing

**Table 2:** Qualitative assessment for antenna deployment testing.

Component	Question	Response
Deployment success	Did the antenna deploy?	YES or NO
Motion quality assessment	Does the antenna motion display jittery behavior?	YES or NO
Deployment completeness	What is the extent of BAC deployment achieved?	PARTIAL or COMPLETE
Structural inspection	Are there any visible cracks on the SMPC sample?	YES or NO *

* Note: If YES, note the location and length of visible cracks using a vernier caliper.

**Table 3:** Quantitative measurements for antenna deployment testing.

Variable	Definition	Unit
Deployment time	Time it took for the antenna to fully deploy	seconds (s)
Activation temperature	Temperature at which the first SMPC release component begins to uncurl	Celsius (°C)

**Table 4:** Qualitative assessment for SMPC-based antenna deployment mechanism.

Bending Angle Configuration	Deployment Success	Motion Quality	Deployment Completeness	Structural Cracks
20	Yes	Partial Non-uniform Motion (SMPC-10)	Complete	None
30	Yes	Partial Non-uniform Motion (SMPC-10)	Complete	None
40	Yes	Partial Non-uniform Motion (SMPC-10)	Complete	None

**Table 5:** Quantitative assessment of the SMPC-based antenna deployment mechanism.

Bending Angle (°)	Deployment Time (s)	Activation Temperature (°C)
20	92 s	60
30	116 s	60
40	122 s	60

## Data Availability

The original contributions presented in this study are included in the article. Further inquiries can be directed to the corresponding authors.

## Abbreviations

The following abbreviations are used in this manuscript:

ABS	Acrylonitrile butadiene styrene
BAC	Bending angle configuration
CF	Carbon fiber-reinforced
DGEBA	Bisphenol A diglycidyl ether
LEO	Low Earth Orbit
PEG	Poly(ethylene glycol)
SMM	Shape memory material
SMP	Shape memory polymer
SMPC	Shape memory polymer composites
*T_g_*	Glass transition temperature
UHF	Ultra high frequency
UV	Ultraviolet
VHF	Very high frequency
